# Differences in Weekly Load Distribution Over Two Euroleague Seasons with a Different Head Coach

**DOI:** 10.3390/ijerph17082812

**Published:** 2020-04-19

**Authors:** Hugo Salazar, Luka Svilar, Ane Aldalur-Soto, Julen Castellano

**Affiliations:** 1 Department of Physical Education and Sport, University of the Basque Country (UPV/EHU), 01007 Vitoria-Gasteiz, Spain; julen.castellano@ehu.eus; 2 Sports Performance Department, Kirolbet Baskonia, 01013 Vitoria-Gasteiz, Spain; luka_svilar@yahoo.com; 3 Lactiker Research group, University of the Basque Country (UPV/EHU), 01006 Vitoria-Gasteiz, Spain; aldalurane@gmail.com

**Keywords:** monitoring, training load, basketball, inertial movement sensor, team sport

## Abstract

The weekly training management and competition loads are important aspects to optimize the performance of professional basketball players. The objectives of the study were (a) to describe the weekly external load (EL), as well as the internal response (IR), of elite basketball players over two consecutive seasons with a different head coach and (b) to compare weekly loads of different competitive densities. The data were collected from 27 elite players from the same team competing in the Spanish first division league (ACB) and EuroLeague during 2017–2018 and 2018–2019 seasons. EL was measured using microsensor technology to determine PlayerLoad values, expressed in arbitrary units (AU). Session rating of perceived exertion (sRPE) was used for IR quantification. Comparisons between the two seasons and of weeks with different competitive densities were made. The inter-week load variability was moderate-high for both seasons. The highest EL values were measured during the weeks with three games (W3) (W3 > W0 > W2 > W1), while the most demanding week for players’ IR was observed during weeks with no competition (W0). Additionally, higher EL (*d* = 0.31) and IR (*d* = 0.37) values were observed in season 2018–2019 compared to 2017–2018. The results obtained in this study contributed new data on the internal and external load required by professional basketball players in weeks with different number of games and showed that different coaching strategies may demand a different external and internal workload in consecutive seasons. Furthermore, the results highlighted the need to carry out an adequate load management program.

## 1. Introduction

Leading European teams usually participate simultaneously in two competitions, a national or regional one (e.g., ACB League in Spain or VTB League in the Baltic countries) and an international one (e.g., EuroLeague, EuroCup or Champions League) during the season. This means that during nine months, a team reaching the play-offs in EuroLeague and domestic finals could play around 80 games in one season. This, together with the frequent travelling all over Europe, could negatively affect the individual and team performance during the games. Sometimes teams have to play at congested periods of four or five games in ten days [1] and therefore, an adequate load distribution strategy is essential [2]. It is important to follow an adequate load distribution strategy together with an appropriate recovery process to have the maximum players available for each game and to reduce injury risk [3].

The number of games played in a week largely determines the accumulation of load on players in team sports during the competitive period [4] and seems to cause fluctuations in the weekly loads that players accumulate [5]. However, according to several authors, the more games played within a week does not necessarily mean a higher load [4,6,7]. Conte et al. [6] reported a greater workload on university players in weeks playing just one game, while Manzi et al. [4] observed higher internal load values when the number of games per week was one instead of two. However, a recent work by Clemente et al. [7] showed that there were no differences between normal (just one official match) and congested (with at least two matches) weeks on perceived exertion. One of the possible reasons for the differences observed among these studies could be due to the length of the period analyzed. Therefore, it would be interesting to carry out studies for longer periods of time (i.e., full seasons), which would avoid the seasonal variability that exists in team sports with a long competitive period [8].

There are numerous methods to quantify training load. To date, the most common ones were focused on the internal load derived from the heart rate, recovery-stress status, immuno-endocrine response or subjective perception of effort [9,10]. The latter is perhaps the most frequent one in basketball due to its accessibility and ease of use both in elite and academy players. To complement the information of the internal response and to have a more global approach of the training load, there is a need to encompass objective indicators of external load [11]. Even though microtechnology is a reliable and effective tool to measure the external load in basketball [12], the scientific information available in elite basketball is scarce [13]. The results obtained in this study will contribute to have reference average values on the accumulated load for each week during a complete season contextualized in elite basketball, as well as to know how the load varies depending on the number of official games played in competitive weeks.

The present study aims to describe the weekly external demands and the internal response from the effort made by players to address two goals: (a) to compare two different training strategies carried out on the same team during two consecutive seasons and (b) to compare weeks with different competitive densities (e.g., number of matches within a week). Our main hypothesis states that a higher competitive density will result in a higher external demand and an increased effort perception by the players.

## 2. Materials and Methods

### 2.1. Subjects

A total of 27 players from the same team participated in the study (age: 24.8 ± 3.2 years; height: 201.3 ± 9.4 cm; weight: 97.7 ± 11.1 kg) during two consecutive seasons (2017–2018 (S17–18) and 2018–2019 (S18–19)) and a time gap of two months between them (off-season period from June to August). The team participated simultaneously in the EuroLeague and the Spanish first division league (ACB) and it had a different head coach in each season. A total of 1041 training sessions and matches were recorded (528 in S17–18 and 513 in S18–19). All the players were familiar with the external and internal load monitoring tools. Players who did not complete 75% of the weekly training duration with the team during a given week were excluded from the analysis. All the players were notified of the purpose, investigation procedures and requirements of the study, as well as the benefits and risks before consenting, in accordance with the Declaration of Helsinki [14]. All the players gave their approval to include their data in the study. This data was analyzed anonymously and with the approval of the Ethics Committee of University of Basque Country (UPV/EHU).

### 2.2. Design

An observational and longitudinal study was carried out in this investigation and the researchers did not interfere at any time in the weekly training plan. The recording of the external load and internal response of the players was monitored every week, including during pre-season and in-season, in two consecutive years (*n* = 43 and *n* = 41 for seasons 2017–2018 and 2018–2019, respectively). Each week was classified according to the number of matches played by the team (W0, W1, W2 and W3 for weeks with 0, 1, 2 and 3 official matches, respectively).

### 2.3. Procedures

At the time when the study was carried out, two different head coaches were in charge of the team in each of the seasons analyzed. The coaches accumulated 17.5 ± 3.5 years of experience in professional basketball. During pre-season, players carried out team strength and power training sessions and played seven friendly matches. During the in-season period, each coach followed his own training strategy. For S17–18, team practices were mainly focused on tactical preparation with non-contact drills together with game-based drills on half court with or without transition (full court was rarely used). Second coach had an opposite strategy that focused on the use of small sided games (e.g., 2 × 2, 3 × 3, 2 × 1, 3 × 2) together with regular 5 × 5 format of play performed on half court with a single transition. Only team practices and matches were included in the analysis. The load derived from strength training (performed in the weight room), individual training, recovery or injury treatment were discarded from the analysis.

The external load was measured by the PlayerLoad^®^ (PL, Catapult, Melbourne, Australia) variable, which determines the mechanical load and has previously been validated in basketball [15], using the Catapult T6 (Catapult Innovations, Melbourne, Australia) portable devices. These devices include an accelerometer, a gyroscope and a magnetometer providing inertial data with a sampling frequency of 100 Hz (dwell time was 0.5 seconds). The devices were placed on the upper back part of the players (interscapular zone) using a specific harness for them. The external load data during training were collected from the beginning of the warm-up until the end of the session, including rest periods, fluid replacement, coaching and other usual training activities. Non-official matches played during pre-season were considered as training sessions. Since the usage of portable devices is not allowed in official matches, the PL during the official games was estimated using individual PL values per played minute (PL/min) and multiplying this value by the time played during the game by each player. The individual PL values were calculated for each player as the average PL value recorded during the seven non-official pre-season games. The average individual PL value for the team was 12.8 ± 1.9 AU/min. Furthermore, 250 AU was added to the estimated PL value in the official games corresponding to the external load during the warm-up period. This value was chosen since a standardized warm-up period of 25 minutes previously recorded during the non-official games was 254.1 ± 24.3 AU. The external load data collected by the devices was downloaded using OpenField software version 1.17 (Catapult, Melbourne, Australia) and exported to an Excel spreadsheet (Microsoft Excel version 16, Microsoft Corporation, Redmond, WA, USA).

Regarding the internal response, the subjective scale of perception of effort CR-10 was used [16]. This scale has been previously validated in team sports [17] and in basketball in particular [10]. As suggested by Singh et al. [18], the players were asked about their perception of the effort fifteen to thirty minutes after the training session or the game. The value of the CR-10 scale was multiplied by the total duration of the session to obtain a variable that encompassed the total training or competition load (sRPE) in arbitrary units (AU).

### 2.4. Statistical Analysis

All the data are presented using the mean and standard deviation (SD). The differences between weeks with different competitive densities (W0, W1, W2 and W3) and between seasons (2017–2018 and 2018–2019) were analyzed using one-way ANOVA and the size of the differences was calculated using the standard differences of the means (Cohen’s d), with its respective limits at 90% of the confidence interval. The interpretation of the effect size (ES) was followed according to Hopkins et al. (2009) [19]: <0.2 is trivial, 0.2–0.6 is small, 0.6–1.2 is moderate, 1.2–2.0 is large and >2.0 is very large. The coefficient of variation (CV, in %) was also calculated dividing the standard deviation by the mean. All analyses were carried out using Microsoft Excel and the statistical analysis software JASP version 0.9.2 (University of Amsterdam, https://jasp-stats.org/, Amsterdam, The Netherlands). The level of significance was set at *p* < 0.05.

## 3. Results

**Figure 1:**
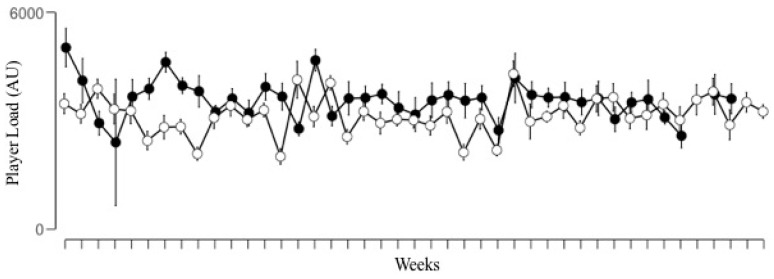
Distribution of the weekly accumulated PlayerLoad in arbitrary units (AU) for the seasons 2017–2018 (o) and 2018–2019 (●).

The mean PL values measured during seasons S17–18 and S18–19 were 3023 ± 855 AU and 3323 ± 1119 AU, respectively, while the average sRPE values observed were 2703 ± 887 AU and 3096 ± 1227 AU, respectively. The variability of the external load and internal response in different weeks, expressed as the CV, was moderate-high for PL (16% and 15%) and sRPE (24% and 19%) in seasons 2017–2018 and 2018–2019, respectively. Figure 1 shows the weekly distribution of the accumulated external load in the two consecutive seasons analyzed. The mean weekly external load measured was higher in S18–19 compared to S17–18, although the effect size was small (ES = 0.31).

**Figure 2:**
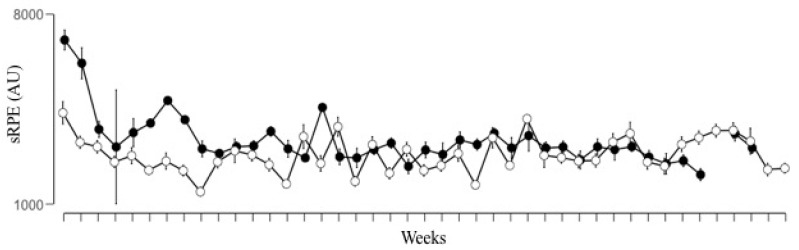
Distribution of the weekly accumulated internal load of subjective perception of effort (sRPE) in arbitrary units (AU) for the seasons 2017–2018 (o) and 2018–2019 (●).

Figure 2 plots the weekly distribution of the accumulated internal response of the players during the two seasons analyzed. The sRPE parameter showed significantly higher values for the season S18–19 than for S17–18, even though the effect size was small (ES = 0.39).

**Figure 3:**
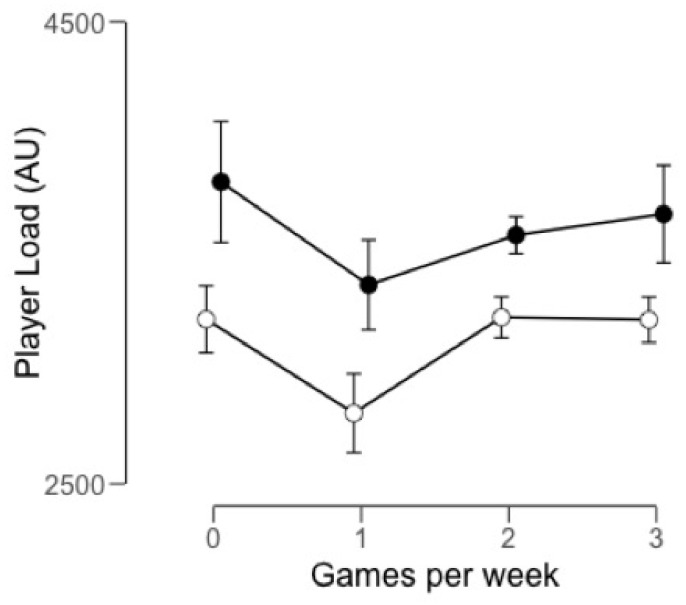
Average PlayerLoad values in arbitrary units (AU) by the number of games played within a week and for the seasons 2017–2018 (o) and 2018–2019 (●). Error bars represent the SD of each of the four groups separately.

The average external load by the number of games played within a week is presented in Figure 3. The number of weeks analyzed for each of the groups was very similar between the seasons 2017–18 and 2018–19: 7 and 6 weeks for W0, 6 and 6 weeks for W1, 22 and 24 weeks for W2 and 8 and 7 weeks for W3, respectively. The weeks without competition corresponded to the pre-season period. During the 2017–2018 season, the weeks with more external load were W2 compared to W1 (ES = 0.57), W3 (ES = 0.02) and W0 (ES = 0.01). 

**Figure 4:**
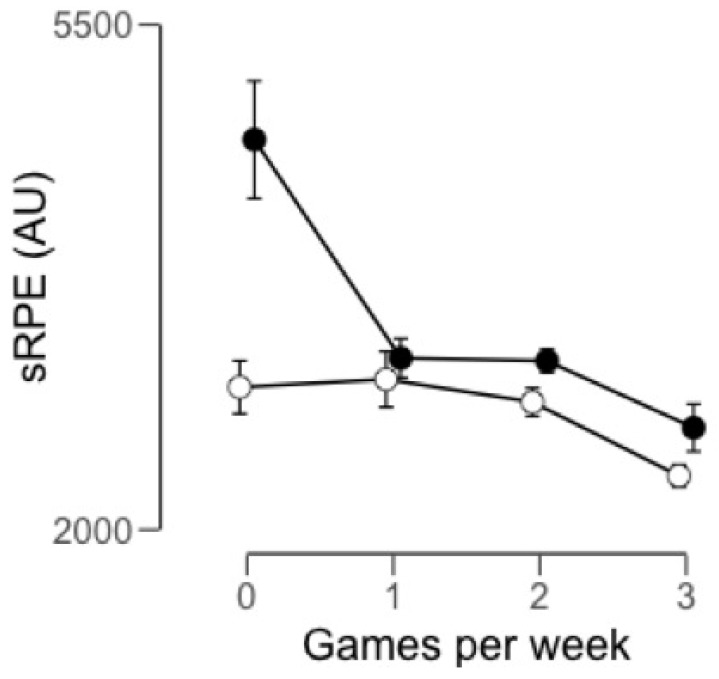
Average session rating of perceived exertion (sRPE) in arbitrary units (AU) by the number of games played within a week and for the seasons 2017–2018 (o) and 2018–2019 (●).

However, the players perceived a greater internal load in W1, although this difference was only significant compared to W3 (ES = 1.09) (Figure 4). Weeks with two games per week for the 2017–2018 season and weeks without a game in 2018–2019 had the largest external load. The players described a greater internal load in the weeks without a pre-season game in 2018–2019. As for the external load of the 2018–2019 season, the highest values were reached at W0 followed by W3 (ES = 0.16), W2 (ES = 0.31) and W1 (ES = 0.49). The same results were obtained for the internal load, with W0 weeks being the ones with the highest AU followed by W1 (ES = 1.35), W2 (ES = 1.70) and W3 (ES = 1.64).

## 4. Discussion

The results obtained in this study showed that the external load and internal response were higher based on the weekly competitive density, although the external load did not increase proportionally when the competitive density was higher. Additionally, the distribution of the external load during training and competition was different for seasons S17–18 and S18–19. The external load measured in S18–19 was significantly higher than in S17–18 and, consequently, the players perceived it as more demanding.

Coaches in all kind of team sports adopt different training strategies regarding quantity (e.g., training duration), quality (e.g., type of drills) or tactical strategies to promote short-term team adaptations and achieve the best possible performance [20]. In spite of the similar competitive context (during both seasons, the team competed in three competitions, two national and one international), the average load accumulated during the weeks, regardless of the number of games per week, was higher for the 2018–2019 season compared to the 2017–2018 season (3323 ± 1119 AU vs. 3023 ± 855 AU). Training drill selection and format of play are therefore key elements that should be considered by coaching staff for practice planning and periodization in weeks with congested competition, in order to maintain the optimal training loads. When designing training drills, variables such as court size, work/rest ratios and level of opposition play an important role in the level of workload that a training task may have. Furthermore, coach encouragement, as well as training formats and court size, may enhance physical demands during trainings [21]. During S18–19, the training strategy used by the coach was based on small-format games with reduced number of players (i.e., 2 × 2, 3 × 3 games) along with regular 5 × 5 games using the full court. This coaching style may explain the higher workload experienced by players during S18–19 as previous research supported that small formats as well as full court drills provide a higher internal and external workload [22,23].

The relationship between external and internal loads has been previously studied in elite basketball showing a positive moderate-high correlation between them [11,24]. The greater variability observed in different weeks for the internal response parameter during both seasons suggests several ideas. First, it is essential to know how a player responds not only to the external demands of training and competition, but also to accumulated fatigue caused by the games on the road [25]. Second, it is also known that the same stimuli could have a different response on players of the same team [2]. Third, the integrated use of external and internal load variables, their relationship and evolution over time could be very interesting in order to control the adjustment of the players to the training and competition load demands [11]. Although previous studies did not show the inter-week variability data in detail [5], the data observed in the present study suggests that the variability in the workload over the weeks of a competitive season in professional basketball is rather high. Avoiding high-load peaks with differences higher than 20%–30% between weeks would reduce the injury risk of the players [26]. Therefore, technical staff should develop adequate strategies to reduce as far as possible the differences in the external load in different weeks, paying special attention to the starting lineup and bench players, thus avoiding defective load management, by default or in excess, which could lead to a loss of form during the season [27].

Elite teams in collective sports (e.g., football, handball and rugby) usually participate in a minimum of two competitions (a national and an international one), which involves playing two games per week. However, this competitive density is even higher in basketball, where the number of games within a week, in many cases, reaches up to three matches in the same microcycle (this happened in 19% of the weeks analyzed in the present study). It is worth to highlight that to play just a single game per week in elite basketball is rather unusual (14% of the weeks in the monitored seasons). Due to the high competitive density of elite basketball, coaches and trainers should design different strategies to optimize the physical condition of the players so that they are able to perform at their highest level in every game [28].

The weekly accumulated internal load values observed for the team in the present study (~3150 AU) were higher than the 2520 AU of the Lithuanian female players [5], similar to the 3200 AU of professional players [7] and lower than the Brazilian female team, where values above 4000 AU were reported [12]. A comparison with collage and Italian professional players showed that the sRPE values were lower than the results shown in this study for W1 and W2 in both seasons [4,6]. This difference may be due to the competitive level of the players analyzed, since the training load in weeks with one or two games for EuroLeague players (the most important European competition) is higher than for other athletes.

In relation to the comparison of the internal load in weeks with different competitive densities, the results obtained in the present study for the season 2018–2019 were similar to those reported by Manzi et al. [4] and Clemente et al. [7], where no differences were found in the effort perceived by players in weeks with one or two games. The lack of consensus on the differences regarding the cumulative load in weeks with different competitive densities may be due to the fact that the data shown by Conte et al. [7] and Manzi et al. [4] were not collected throughout a whole season, but only for a specific period of time within a season. Teams use different load strategies during a season depending on the importance of the moment [4], and this would explain the different results obtained among the studies. Finally, it should be noted that, as in other team sports, a greater external load and internal response was accumulated during the first weeks of training (pre-season), as it is a key period to physically prepare the players [29]. This pattern was repeated in the two seasons analyzed in this study, especially in season 2018–2019, with values exceeding the weeks with official competition [30].

This study is not without its limitations, which should be considered when extrapolating the data to other teams or different competitive levels. Firstly, although the team monitored may be representative of a top-level club in the European arena, only one team and the training strategies of two coaches during two consecutive seasons were analyzed. As a greater number of in-depth studies on load management are available, the training and competition load values measured in this work could be used as a guideline for professional basketball teams. Secondly, the monitoring of the training load was only performed based on global indicators of external load (PL) and internal response (sRPE). These indicators only provide a general idea of training and competition, so for future studies it would be interesting to include representative variables of movement (e.g., distances traveled) and inertia (e.g., acceleration, changes of direction, jumps and impacts), as well as internal adaptation variables of the players, such as heart rate and hormonal markers [31]. Finally, it is important to note that future research should focus on a deeper analysis of training drills used by head coaches, as it may help practitioners to better understand periodization, especially the variability between the weeks with different number of games.

## 5. Conclusions

This study indicates that the external load and internal response values of the weekly workload could be used as a guideline in elite basketball during a complete competitive season. Additionally, different head coaches could manage weekly loads with different magnitudes regardless of the number of games per week. The distribution of the workload over the weeks is based on the competitive density of a microcycle, which should be considered by coaching staff in order to (a) implement recovery strategies, mostly in periods with a greater number of games per week [32], (b) manage training workload in weeks with no competition as the players perceived higher levels of tiredness on these weeks and (c) provide enough rest to players with high volume of minutes during games. Additionally, before competition periods starts, players should be trained in order to achieve high chronic weekly loads. This would enhance players’ fitness level and protect players from possible injuries in congested competitive periods where spikes in weekly load can happen [33].
